# Demineralization of Osseous Structures as Presentation of a Rare Genetic Disorder That Is Associated With a High Rate of Mortality

**DOI:** 10.1155/crie/6063059

**Published:** 2024-12-12

**Authors:** Adeeba Afrah, Michael A. Finkel, Carolina Fonseca, Marianne Tomiyoshi Asato, M. Susan Jay, Athina Pappas, Shashikala B. Gowda, Allison Jay

**Affiliations:** ^1^Department of Pediatrics, Division of Pediatric Pulmonology, University of Michigan, Medicine, Ann Arbor, Michigan, USA; ^2^Division of Genetic, Genomic and Metabolic Disorders, Department of Pediatrics, University of Michigan, Ann Arbor, Michigan, USA; ^3^Department of Pediatrics, Division of Pediatric Hospital Medicine, University of San Francisco, San Francisco, California, USA; ^4^Department of Genetics, Advent Health, Orlando, Florida, USA; ^5^Department of Pediatrics, Medical College of Wisconsin, Milwaukee, Wisconsin, USA; ^6^Henry Ford St. John Hospital, Detroit, Michigan, USA

## Abstract

**Objectives:** Describe the details of the clinical presentation, diagnostic challenges, and management of a female neonate with neonatal severe hyperparathyroidism (NSHPT).

**Methods:** This case report was developed from a retrospective chart review. The female infant was born to consanguineous parents—first cousins, with multiple prenatal concerns, including gestational diabetes, intrauterine growth restriction, polyhydramnios, and suspicion of a hypoplastic left atrium on prenatal echocardiogram (ECHO). Following a planned C-section at 37 weeks gestation, the neonate exhibited moderate respiratory distress with subcostal retractions. On physical examination, craniotabes, a bell-shaped chest, and a continuous machinery-type murmur were noted.

**Results:** Evaluation at birth revealed a large Patent Ductus Arteriosus and significant demineralization of skeletal structures with atypical rib morphology. Lab work at 24 h of life (HOL) showed elevated serum calcium level (14.3 mg/dL), ionized calcium—iCal (2.32 mmol/L), and normal 25-OH Vitamin D (54.2 ng/mL). A comprehensive skeletal survey uncovered generalized osteopenia, metaphyseal lucencies, and evidence of healing fractures. Repeat lab work at 43 HOL, showed serum calcium of 18.0 mg/dL, iCal 2.67 mmol/L, and elevated parathyroid hormone (PTH) of 2116 pg/mL. Diagnosis of NSHPT was established based on laboratory findings. Molecular testing confirmed a homozygous variant (c.1744T >A; p.Cys582Ser) in the calcium-sensing receptor (CaSR) gene which confirmed the diagnosis of NSHPT. NSHPT, a rare genetic disorder associated with high mortality rates, is often caused by inactivating CaSR gene variants. The patient's family history revealed a strong correlation with familial hypocalciuric hypercalcemia (FHH), a benign condition associated with asymptomatic hypercalcemia, normal to minimally elevated parathyroid level, and hypocalciuria, it is caused by heterozygous inactivating mutations in the CaSR gene. Treatment of NSHPT typically involves total or subtotal parathyroidectomy; however, initial medical intervention is often necessary. In this case, the neonate underwent medical treatment with calcitonin, furosemide to help facilitate renal clearance of calcium, and intravenous fluids before a successful parathyroidectomy.

**Conclusions:** This case accentuates the importance of considering rare genetic disorders in neonates with complex clinical presentations and affirms the need for comprehensive counseling and education, particularly in consanguineous parents, to address familial implications and guide appropriate interventions.

## 1. Presentation

At 37 weeks gestational age, a female neonate was delivered via planned C-section due to prenatal concerns including maternal gestational diabetes, intrauterine growth restriction, polyhydramnios, and a fetal echocardiogram (ECHO) showing hypoplastic left atrium with a mildly dilated right atrium. Before birth, admission to the Neonatal Intensive Care Unit (NICU) was arranged for cardiac assessment and further evaluation. Upon examination, the neonate's APGARs were 8 and 9 at 1 and 5 min, respectively. The neonate appeared ill, exhibiting moderate respiratory distress with subcostal retractions, craniotabes, and a bell-shaped chest. After the initial assessment, the infant was placed on noninvasive (NIV) positive pressure ventilation (PPV) for respiratory compromise. Vital signs were a temperature of 36.9°C, respiratory rate of 57, heart rate of 139, and oxygen saturation of 99%–100% on FiO2 of 30%. General craniotabes were appreciated. A cardiac exam revealed a continuous machinery-type murmur. Pulses were easily palpable, symmetric, and synchronous. The abdominal exam was unremarkable. Neurological evaluation revealed extended posture and mildly decreased muscle tone. The musculoskeletal examination was significant for mild bowing of the bilateral femurs, normal muscle bulk, and no limb length discrepancy. Careful skin evaluation revealed no erythema, nodules, or evidence of subcutaneous fat necrosis.

**Figure 1:**
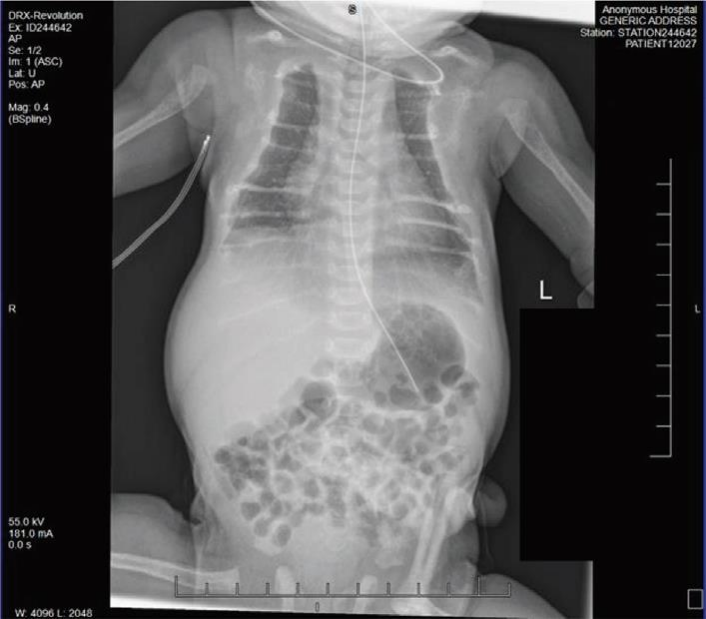
Bell-shaped chest with significant demineralization of the osseous structures and atypical rib morphology consistent with healing fractures.

**Figure 2:**
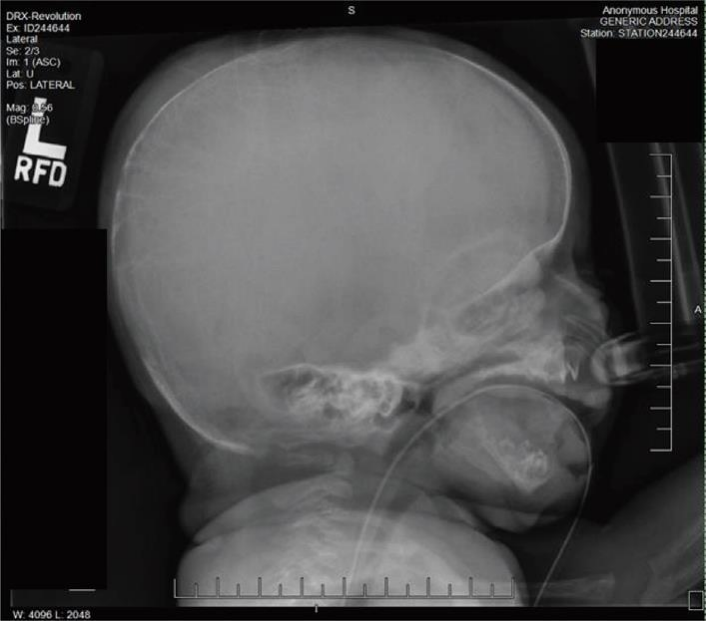
Bone demineralization of the skull, prominence of the soft tissue in the scalp parietal region.

**Figure 3:**
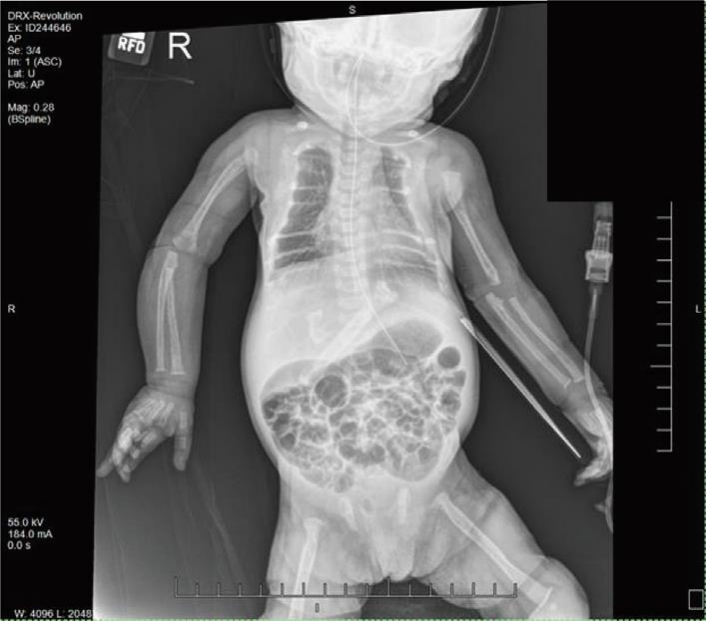
Generalized osteopenia, metaphyseal radiolucencies, multiple osseous irregularities, and mild bowing of the right proximal humerus suggesting healing fracture(s).

Subsequent echocardiography disclosed a large patent ductus arteriosus, elevated right ventricular systolic pressure, mildly dilated right atrium, but otherwise structurally normal heart. Chest X-ray revealed significant demineralization of the osseous structures and atypical rib morphology with a bell-shaped appearance of the chest (Figure 1). Following this, a skeletal survey was performed that disclosed generalized osteopenia, with prominence of the soft tissue in the scalp parietal region as well as metaphyseal lucencies and mild bowing of the right proximal humerus suggesting healing fracture(s) (Figures 2 and 3).

Laboratory results at 24 h of life revealed: a serum calcium level of 14.3 (normal range 8.4–10.6 mg/dL), ionized calcium (iCal) of 2.32 (normal range 1–1.5 mmol/L), Vitamin D 25-hydroxy level of 54.2 (normal range 31–80 ng/mL), and parathyroid level of 2116 (normal range: 15–65 pg/mL) (Table 1). The patient was treated with IVF at 1.5 maintenance, furosemide, and calcitonin. Transfer to a tertiary medical center was arranged and comprehensive diagnostic testing revealed the underlying condition.

## 2. Discussion

### 2.1. Differential Diagnosis

The initial differential diagnoses based on the chest X-ray and skeletal survey findings included: osteogenesis imperfecta, skeletal ciliopathies (e.g., Jeune syndrome), and other skeletal dysplasias, which manifest with the characteristic osseous demineralization evident in imaging. Congenital syphilis, though less commonly associated with this severity of bony changes, remains an important consideration due to the neonate's respiratory distress and the potential for systemic involvement affecting bone development. Additionally, rare metabolic bone diseases such as hypophosphatemia, and parathyroid mediated disease (sporadic and inherited causes of primary hyperparathyroidism or dysfunction of the calcium-sensing receptor [CaSR]) warrant attention, particularly when accompanied by significantly elevated calcium and parathyroid hormone (PTH) levels, as observed in this case. While nutritional rickets secondary to maternal vitamin D deficiency are a consideration, their likelihood is diminished in developed nations where nutritional deficiencies are less prevalent, especially in the absence of significant concerns regarding maternal malnourishment preceding delivery.

### 2.2. Actual Diagnosis

Laboratory testing was consistent with a diagnosis of neonatal severe hyperparathyroidism (NSHPT). In cases presenting with neonatal osseous demineralization and bone deformities, diagnosing the underlying cause of a suspected skeletal dysplasia requires thorough clinical, imaging, biochemical, and molecular evaluation, and helps inform appropriate neonatal management and family counseling.

Following NICU admission, comprehensive laboratory tests were obtained and revealed a markedly elevated PTH level, measuring 2116 pg/mL, well beyond the normal range of 12–88 pg/mL. Radiographic skeletal survey findings of generalized osteopenia, accompanied by characteristic skeletal anomalies including short ribs and irregular mineralization, along with evidence of healing fractures involving multiple extremities bilaterally further supported the diagnosis (Figures 1–3).

Subsequent ultrasound examination of the neck failed to detect any evidence of parathyroid adenoma. Molecular testing confirmed the suspected diagnosis, identifying a homozygous variant in *CASR* (c.1744T >A; p.Cys582Ser). This missense variant is listed in ClinVar (https://www.ncbi.nlm.nih.gov/clinvar/variation/853442) as likely pathogenic, as it has previously been reported in individuals with disease and has not been reported in healthy population databases. Our case offers further support that this variant is disease causing.

Upon disclosure of the diagnosis to the family, pertinent family history surfaced. The parents volunteered that their union was consanguineous. The patient's father also disclosed a familial pattern of parathyroidectomy and familial hypocalciuric hypercalcemia (FHH) among paternal relatives including him; although biochemical laboratory data from these individuals were not available. Further genetic analysis revealed the father to be heterozygous for the familial *CASR* variant, shedding light on the hereditary nature of the neonate's condition. At present, genetic testing for the patient's mother is pending, emphasizing the importance of familial screening, and genetic counseling about inheritance and recurrence risk of NSHPT in future offspring.

### 2.3. The Condition

NSHPT is a rare but serious, autosomal recessive condition presenting in children within the first 6 months of life[1, 2]. It is characterized by abnormally high levels of PTH and calcium in newborns. Common presentations include lethargy, poor feeding, vomiting, dehydration, constipation, failure to thrive, hypotonia, respiratory distress, skeletal demineralization, and even seizures [1, 2]. More than 200 cases have been reported in the literature with variable disease manifestations and severity [3]. The CaSR is a G protein-coupled receptor encoded by *CASR* (OMIM#601199) located on chromosome 3q13.3-q21.1 expressed in the parathyroid gland and renal tubules and helps regulate calcium homeostasis in conjunction with receptor binding [4–6]. In addition, CaSR helps control PTH synthesis and secretion in response to changes in serum calcium and urinary reabsorption/excretion in the kidney [4–6].

Several genetic mutations in *CASR* lead to distinct phenotypes. Both loss-of-function and gain-of-function mutations have been documented. Loss-of-function mutations (as seen in our patient) can manifest as either familial FHH or NSHPT [1, 2]. Patients with FHH, due to heterozygous pathogenic variants, typically do not require treatment but should be educated on their diagnosis to avoid future misdiagnosis and unnecessary workup or treatment. The majority of patients with NSHPT are identified as having homozygous or compound heterozygous pathogenic variants of *CASR* and many patients have a family history of hypocalciuric hypercalcemia [1, 2].

### 2.4. Treatment and Management

Treatment of NSHPT is total or subtotal parathyroidectomy, but most patients require initial medical intervention to stabilize calcium levels [5]. The initial management includes hyperhydration to promote saline diuresis with or without the use of loop diuretics to prevent fluid overload and optimize renal calcium clearance. Bisphosphates and new agents, such as cinacalcet, offer alternative modalities to bridge the patient to parathyroidectomy when the patient is more stable [7, 8]. Cinacalcet, a calcimimetic agent, has shown benefit in the management of NSHPT [7, 8]. By targeting CaSR on the parathyroid gland, cinacalcet can effectively lower PTH levels and thereby normalizing serum calcium concentrations.

### 2.5. Patient Course

Considering the infant's small size and respiratory insufficiency, the initial plan was to medically manage her hypercalcemia. Treatment commenced with hyperhydration via IV fluids and furosemide administration to enhance renal calcium clearance. The patient's daily fluid balance was closely monitored in the ICU setting to ensure she did not experience iatrogenic dehydration. Additionally, the infant was prescribed calcitonin and cinacalcet.

At ~6 weeks of age, she underwent a parathyroidectomy procedure, with one-half of a gland reimplanted onto the sternocleidomastoid muscle. No acute orthopedic surgical interventions were undertaken during her hospitalization for her fractures. Her electrolytes were closely monitored throughout her hospitalization.

The infant's rib deformities and demineralization contributed to respiratory distress that necessitated the initiation of NIV PPV following birth. Ventilator dependence persisted until ~5 months of age when the patient achieved stability while using low-flow nasal cannula (LFNC) at a rate of 0.03L O_2_. She remained on supplemental oxygen and subsequently developed pulmonary hypertension that was treated with sildenafil therapy. Throughout her hospitalization, multiple ECHOs were performed to closely monitor her elevated pulmonary pressures. As a result of prolonged intubation, the infant developed oral aversion, necessitating the placement of a G tube; she was started on a low calcium-containing formula.

Discharge from the NICU occurred at 7 months of age, with prescriptions for sildenafil and Calcitriol. Detailed instructions were provided to the parents regarding outpatient follow-up.

### 2.6. Lessons for the Clinicians

• FHH increases the risk of NSHPT.
• NSHPT is linked to both homozygous and compound heterozygous germline-inactivating variants of *CASR* encoding CaSR.
• Timely detection of NSHPT is essential to improve the prognosis.
• Neonatal hyperparathyroidism should be considered in infants presenting with osteopenia, a bell-shaped chest, and hypomineralization.
• Conduct tests for Calcium, phosphorus, PTH, alkaline phosphatase, and Vitamin D in infants exhibiting signs of bone hypomineralization.
• Treatment options may include hyperhydration with or without loop diuretics, cinacalcet, and surgical parathyroidectomy. Given the potential impact of severe hypercalcemia on myocardial depolarization, monitoring cardiac function is crucial.

## Figures and Tables

**Table 1:** Serum Ca, ionized Ca, vitamin D, and parathyroid level.

Analyte	At 24 HOL	At 43 HOL
Serum Ca (normal 8.4–10.6 mg/dL)	14.3	18.0
Ionized Ca (normal 1–1.5 mmol/L)	2.32	2.67
Vitamin D (normal 31–80 ng/mL)	54.2	—
Parathyroid level (normal 15–65 pg/mL)	—	2116

Abbreviation: HOL, hours of life.

## Data Availability

All data underlying the results are available as part of the case report and no additional source data are required.
